# Successful control of the first carbapenem-resistant *Klebsiella pneumoniae* outbreak in a Chinese hospital 2017–2019

**DOI:** 10.1186/s13756-020-00757-y

**Published:** 2020-06-22

**Authors:** Jiaying Zhu, Qi Li, Xiaoxia Li, Jianbang Kang, Yan Song, Junli Song, Donghong Yin, Jinju Duan

**Affiliations:** 1 Department of Pharmacy, school of Pharmacy, Shanxi Medical University, Taiyuan, Shanxi People’s Republic of China; 2 Department of Pharmacy, Baotou Hospital of Traditional Mongolian and Chinese Medicine, Baotou, Inner Mongolia People’s Republic of China; 3 Department of Pharmacy, Second Hospital of Shanxi Medical University, No 382, Wuyi Road, Xinghualing District, Taiyuan, Shanxi People’s Republic of China

**Keywords:** Carbapenem-resistant *Klebsiella pneumoniae*, Infection control, NDM, KPC, Phenotypic detection, Transmission

## Abstract

**Background:**

Carbapenem-resistant *Klebsiella pneumoniae* (CRKP) is considered as a serious global threat. CRKPs occurred only sporadically in the Second Hospital of Shanxi Medical University. Our study aimed to investigate and control the first outbreak of CRKP in our hospital occurred between October 2017 and August 2019.

**Methods:**

The antimicrobial stewardship (AMS) workers have been implemented control measures properly. Clinical and epidemiological data were retrospectively collected from medical records. Carbapenemase genes were detected by modified carbapenem inactivation method (mCIM) test and the EDTA-modified carbapenem inactivation method (eCIM) test. Resistance genes were identified by polymerase chain reaction (PCR) and sequencing. Genetic relatedness was studied by multilocus sequence typing (MLST).

**Results:**

During the outbreak, 31 patients were infected with CRKP isolates. 20 (64.5%) patients were infected with KPC-2 and/or NDM-1 producing *K. pneumoniae*. Mostly MLST-sequence types belonged to ST11 (21/31). The outbreak was two major *K. pneumoniae* clusters present in epidemiologically linked patients.

**Conclusions:**

Setting up AMS workers is potentially a highly efficient strategy for the successful control of the outbreak. A multimodal and multidisciplinary infection control strategy proved to be crucial. The emergence of CRKP in our hospital emphasizes the importance of continuous monitoring of these isolates, which helps to limit the spread of CRKPs and improve the level of management.

## Introduction

*Klebsiella pneumoniae* is one of the leading causes of hospital-acquired infections globally. Carbapenems are widely used for the treatment of serious infections caused by multidrug resistant (MDR) *Enterobacteriaceae* such as AmpC β-lactamases and extended spectrum β-lactamases (ESBLs) [1]. However, in recent years, widespread use of carbapenems have accelerated the growth of resistant strains in different regions [2]. The problem of antimicrobial resistance is highlighted by a recent increase of carbapenem-resistant *K. pneumoniae* (CRKP), which has largely been driven by the emergence and spread of mobile genetic elements carrying carbapenem resistance genes including either class B metallo-betalactamases (IMP, VIM, NDM) or classes A (KPC) and D (OXA-48) serine carbapenemases [3, 4]. KPC-producing strains, first reported in 1996 [5], are considered as one of the most common carbapenemases globally [3]. In China, KPC-producing *K. pneumoniae* strains have been identified since 2004 in Zhejiang Province [6] and have became endemic in many hospitals, with most of them harbouring blaKPC-2 allele [7]. In addition, blaNDM-1 is now considered to be endemic in the Indian subcontinent [8] and reports of NDM-1-producing *Klebsiella* from hospital- and community-acquired infections elsewhere indicate global dispersion [9].

A total of 44 hospitals were included in the China Antimicrobial Surveillance Network (CHINET, www.chinets.com) in 2018. Most of the hospitals included are the largest tertiary-care teaching hospitals in each province or city which represent 26 provinces or cities. These provinces or cities have a population of about nine hundred million. It has reported that Carbapenem resistance among *K. pneumoniae*, especially cultured from cerebrospinal fluid, increased significantly from 18.6 to 64.1% in 2018 [10]. From 2005 to 2018, the resistance rates of *K. pneumoniae* to imipenem and meropenem were increased from 3.0 to 25%, 2.9 to 26.3%, respectively [10]. Dissemination of the CRKP strains is facilitated by inadequate infection prevention and control practice in healthcare settings, uncontrolled or poorly controlled antimicrobial use, sewage water treatment and general sanitation. The spread of such strains is associated with high mortality rates, limited treatment options and rapid dissemination of successful bacterial clones in the hospital setting. The occurrence of the above conditions can be effectively reduced by taking strict infection control measures [11]. In this report, we describe what we believe to be the first outbreak of CRKP in our hospital, which occurred from October 2017 to August 2019. Our aims were to assess the antimicrobial resistance phenotypes, the epidemiology, clinical features, outcomes and the challenges faced in controlling CRKP during the 23 months from October 2017 to August 2019 in a tertiary-care teaching hospital in Shanxi, China.

## Materials and methods

### Data collection

The Second Hospital of Shanxi Medical University is a 2076-bed tertiary-care teaching hospital. A total of 31 non-duplicated CRKP strains were isolated and identified from October 2017 to August 2019. All clinical and epidemiological data were retrospectively reviewed. All isolates in this study were defined as resistant to one of meropenem, imipenem or ertapenem. A CRKP-positive case was defined as any patient infected or colonized with CRKP. All isolates were identified by VITEK-2 Compact system (BioMerieux Italia S.p.A) and matrix-assisted laser desorption ionization time-of-flight mass spectrometry (Bruker Daltonik, Bremen, Germany).

### Antimicrobial susceptibility testing

Antimicrobial susceptibility was evaluated by the agar dilution and microdilution methods at the Second Hospital of Shanxi Medical University according to the Clinical and Laboratory Standards Institute (CLSI) guidelines [12], and the results were interpreted according to CLSI categories and minimum inhibitory concentration (MIC) breakpoints. The breakpoint of tigecycline for *K. pneumoniae* was based on the US Food and Drug Administration standard. *Escherichia coli* ATCC 25922 were used as quality control standards for antimicrobial susceptibility testing.

### Investigation of resistance mechanisms

To use the combined application of modified carbapenem inactivation method (mCIM) test and the EDTA-modified carbapenem inactivation method (eCIM) test for discriminating between serine- and metal-dependent (such as metallo-β-lactamases (MBLs) carbapenemases) [12, 13]. For 31 CRKP strains, polymerase chain reaction (PCR) was used to detect genes encoding carbapenemases (bla_KPC_, bla_NDM_, bla_GES_, bla_IMP_, bla_VIM_, bla_SIM_, and bla_OXA-48_), ESBLs, and AmpC β-lactamases (bla_CTX-M-1,3,10-12,15_, bla_CTX-M-2,4–7,Toho-1_, bla_CTX-M-9,13-14,16–19,Toho-2_, bla_ACT_, bla_DHA_, and bla_CMY_), as previously described [5, 14–18]. The colistin resistance gene mcr-1 was also detected by PCR, as previously described [19]. PCR products were purified with a QIAquick PCR Purification Kit (Qiagen, Valencia, CA, USA) and sequenced by Sanger sequencing on an ABI PRISM 3730XL system (Applied Biosystems, Foster City, CA, USA).

### Multilocus sequence typing (MLST)

Multilocus sequence typing (MLST) was performed by amplifying the internal fragments of seven *K. pneumoniae* housekeeping genes according to the MLST website (https://bigsdb.pasteur.fr/). A phylogeny was showed by using the seven concatenated MLST gene sequences of all 31 strains. We constructed a refined maximum likelihood phylogeny by MEGA7 to study these isolates in greater detail.

### Infection control program

In order to control the outbreak of infection, we have set up a working group on the antimicrobial stewardship (AMS) in August 2018. The AMS workers consisted of hospital administrators, health directors, physicians, nurses, clinical pharmacists, a microbiologist, an environmental cleaning staff and staffs from the infection management department, infectious diseases department and computer center department.

During the outbreak, strict infection control procedures were put in place including a) Rapid communication between microbiological laboratories and AMS workers was established to ensure early warning and timely sharing of any pertinent epidemiological information, access to online results of microbiological testing was already routinely available; b) Infection control specialists and clinical pharmacists put forward suggestions on rational drugs use in time and staffs underwent training courses. All CRKP carriers were isolated in a single room with strict contact precautions and flagged in the hospital information system (HIS); c) The AMS workers enhanced chlorhexidine disinfection of the patients’ room at least three times a day and implemented surveillance cultures of the hand and environment. The AMS team held meetings regularly for update the outbreak situation, the implemented measures and proposed future actions; d) All contact patients are systematically subjected to rectal and pharyngeal screening once a week; e) After CRKP-positive cases were discharged, the surroundings were cleaned in depth using a 500-ppm chlorine solution. High-touch surfaces such as door handles, bedside lockers and chairs and bed rails were emphasized by the hospital hygiene nurse manager for cleaning to reduce cross-transmission. Infection control staffs followed up the measures; f) When the affected patients transfer to other hospitals, these hospitals should be forewarned.

## Results

### Epidemiological investigation of the outbreak

**Fig. 1:**
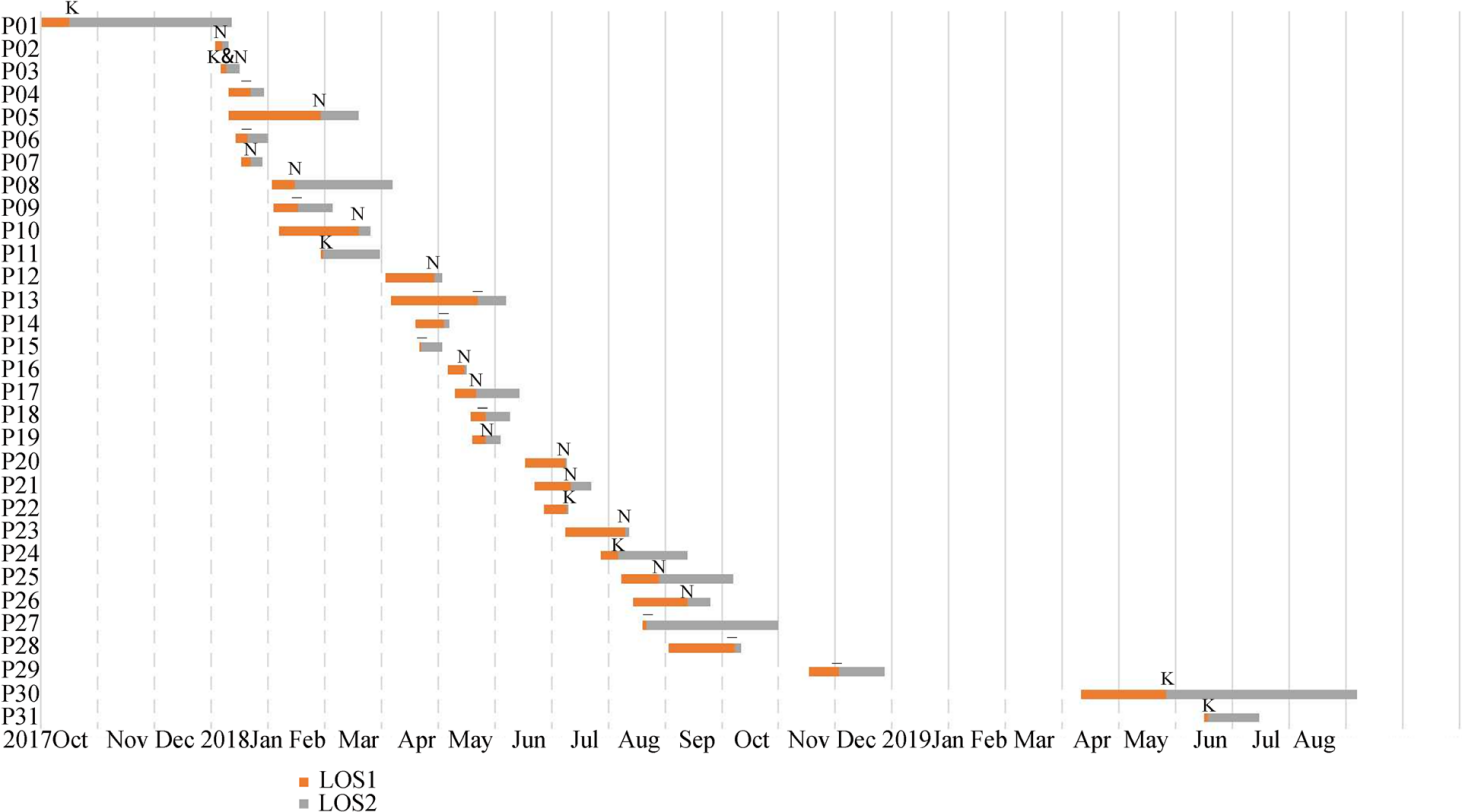
Timeline of events during the outbreak of carbapenemase-resistant *K. pneumoniae*. Dates are given as month. LOS1: Length of stay until first detection; LOS2: Length of stay after first detection; K: KPC; N: NDM; K&N: KPC and NDM; −: no-KPC and no-NDM

**Fig. 2:**
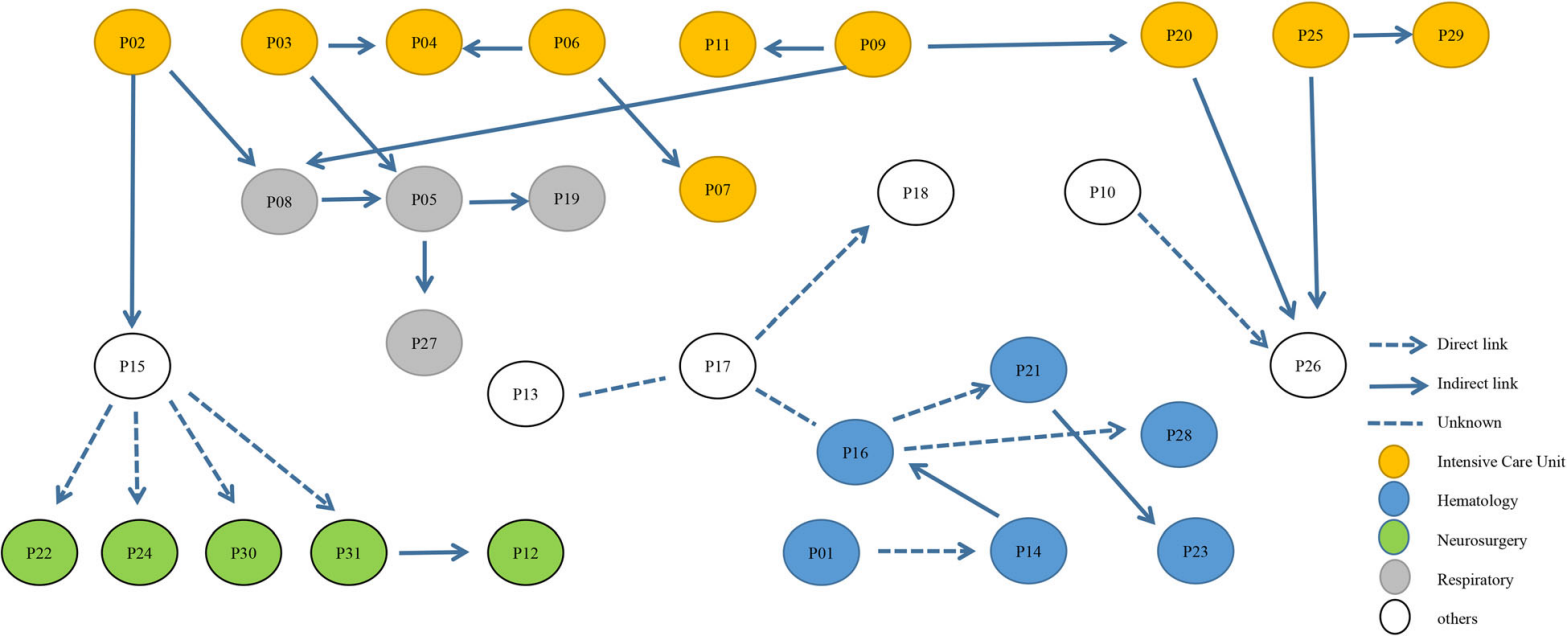
Epidemiological links between CRKP patients (*n* = 31 cases). Nodes represent cases (1–31) and color represent department when infection. Arrows indicate epidemiological link between cases, directly (patients in the same ward) or indirectly (share common room, environmental source or via undetected intermediate patient)

Until 2018, CRKPs occurred only sporadically. During October 2017, CRKP was detected from one patient in Hematology ward, who was hospitalized because of PH-positive acute lymphoblastic leukemia with lack of granulocytes. The patient did test positive for CRKP from fecal culture which was turn out to be colonization. Two month later, the patient gradually tests positive from both sputum and blood again. That patient was defined as patient 01 (Fig. 1). After that in January 2018 the two index patients in the intensive care unit (ICU) were detected, whom defined as patient 02 and 03, neither had a history of hospitalization nor travel in a foreign country. Then three other patients were test positive for CRKP in 2 days and all of them were in the ICU, with no signs of infection or colonization on admission. They were in individual beds in the same room but had overlapping nurses, physicians, and respiratory technicians, they also shared thermometers (disinfection of alcohol cotton swabs) and stethoscopes before isolation precautions were ordered. At the affected wards new CRKP strains were isolated, five in January and three in February (Table 1). Some of them were in direct contact with patient 02 or 03 in the ICU, while others who came into contact with ICU of patient 02 or 03 were subsequently transferred to regular wards (Fig. 2). Some of the patients transferred to the regular wards were in the same room. In a few new CRKP-positive cases there was no direct contact with infected patients, however, they were in adjacent rooms and were cared for by the same doctor, nurse or environmental cleaning staff (Fig. 2).

**Table 1:** Epidemiological and clinical data of the 31 patients infected with CRKP during the outbreak

										Carbapenemase					
ID	Sex	Age(y)	APACHE II	Department when infection	Clinic samples	Outcome	CIM	eCIM	MLST	KPC	NDM	Other	ESBL	AmpC	mcr-1
P01	F	56	14	Hematology	Blood	Death	I	–	ST147	–	–	–	+	–	–
P02	M	62	32	ICU	Respiratory secretions	Discharge	+	+	ST1	–	NDM-1	–	–	–	–
P03	F	78	12	ICU	Respiratory secretions	Death	+	+	ST11	KPC-2	NDM-1	–	–	–	–
P04	M	88	20	ICU	Respiratory secretions	Death	+	+	ST11	–	–	–	–	–	–
P05	M	52	26	Respiratory	Respiratory secretions	Discharge	+	+	ST11	–	NDM-1	–	–	–	–
P06	M	48	20	ICU	Respiratory secretions	Death	+	+	ST11	–	–	–	–	–	–
P07	M	40	26	ICU	Respiratory secretions	Death	+	+	ST11	–	NDM-1	–	–	–	–
P08	M	54	20	Respiratory	Respiratory secretions	Death	+	+	ST11	–	NDM-1	–	–	–	–
P09	M	54	26	ICU	Respiratory secretions	Death	+	+	ST11	–	–	–	–	–	–
P10	F	85	14	Gastroenterology	Secretions	Discharge	+	+	ST11	–	NDM-1	–	–	–	–
P11	M	56	25	ICU	Respiratory secretions	Discharge	+	–	ST11	KPC-2	–	–	+	+	–
P12	M	76	24	Neurosurgery	Cerebrospinal fluid	Death	+	+	ST11	–	NDM-1	–	–	–	–
P13	M	44	5	General Surgery	Pus	Discharge	+	+	ST11	–	–	–	–	–	–
P14	M	30	6	Hematology	Respiratory secretions	Death	–	–	ST15	–	–	–	–	–	–
P15	M	90	15	Cardiothoracic Surgery	Blood	Discharge	+	+	ST11	–	–	–	–	–	–
P16	M	62	10	Hematology	Respiratory secretions	Discharge	+	+	ST3744	–	NDM-1	–	–	–	–
P17	M	66	13	Nephrology	Respiratory secretions	Discharge	+	+	ST11	–	NDM-1	–	–	–	–
P18	M	62	8	Rheumatology	Blood	Discharge	+	+	ST11	–	–	–	–	–	–
P19	F	71	7	Respiratory	Respiratory secretions	Discharge	+	+	ST11	–	NDM-1	–	–	–	–
P20	M	42	7	ICU	Blood	Discharge	+	+	ST11	–	NDM-1	–	–	–	–
P21	M	57	9	Hematology	Respiratory secretions	Discharge	+	+	*	–	NDM-1	–	+	–	–
P22	M	70	29	Neurosurgery	Respiratory secretions	Death	+	–	ST11	KPC-2	–	–	+	–	–
P23	F	44	18	Hematology	Blood	Discharge	+	+	ST690	–	NDM-1	–	–	–	–
P24	F	76	11	Neurosurgery	Cerebrospinal fluid	Discharge	+	–	ST11	KPC-2	–	–	+	–	–
P25	M	45	10	ICU	Blood	Discharge	+	+	ST11	–	NDM-1	–	–	–	–
P26	F	38	5	Orthopedics	Respiratory secretions	Discharge	+	+	ST11	–	NDM-1	–	–	–	–
P27	M	95	16	Respiratory	Respiratory secretions	Discharge	+	+	ST784	–	–	–	–	–	–
P28	F	68	18	Hematology	Blood	Discharge	–	–	ST7	–	–	–	–	–	–
P29	F	85	7	ICU	Secretions	Death	+	+	ST524	–	–	–	–	–	–
P30	M	67	22	Neurosurgery	Cerebrospinal fluid	Discharge	+	–	ST307	KPC-2	–	–	–	–	–
P31	M	76	3	Neurosurgery	Cerebrospinal fluid	Discharge	+	–	ST11	KPC-2	–	–	+	–	–

*APACHE II* Acute Physiology and Chronic Health Evaluation II, *ST* sequence type, *ICU* intensive care unit, *mCIM* modified carbapenem inactivation method, *eCIM* EDTA-modified carbapenem inactivation method, *MLST* multilocus sequence typing, *ESBLs* extended spectrum β-lactamases; *: data not publish; —: negative

From the October 2017 to August 2018, a total of 26 patients with KPC-2 and/or NDM-1 CRKP were found in ten different wards (including ICU), all with an epidemiological connection to the outbreak. As this was now considered as an outbreak, hospital infection management department were notified. The AMS workers ensuring that these measures mentioned in the methods were implemented correctly. With all precautions in place, the outbreak began to wane in September 2018 with only few newly infected patients and no additional spread in the most affected wards (Fig. 1 and Table 1). The outbreak essentially stopped in December 2018. Due to the successful control of the outbreak we are now back to epidemiological stage before the outbreak with sporadic CRKP occurrence. From May to June 2019, CRKP with KPC was detected in two newly CRKP-infected patients with indirect epidemiological connection to the outbreak (Figs. 1 and 2).

A total of 31 patients were affected. These patients did not have a history of travel outside of China, but most of them had frequent hospitalizations in Shanxi province. In 17 (54.8%) patients CRKPs were isolated from respiratory secretions. In seven (22.6%) patients CRKPs were isolated from blood cultures, in four (12.9%) from cerebrospinal fluid, in two (6.5%) from the secretions and in one case (3.2%) from Pus (Table 1).

The age range of the patients was from 30 to 95 years, with an average age of 62.4 years, and 71.0% were male. The average score on the Acute Physiology and Chronic Health Evaluation II (APACHE II) index of these patients was 15.4 (Table 1).

The average number of days spent in hospital was 38.0 days. The average number of days for infection development after first detection of CRKP from patients was 21.0 days, and the median was 12.0 days (Fig. 1). A total of 45.2% of CRKP patients received Carbapenem-including treatment in the 90 days before the strains isolated. Among all the patients in this study, 11 (33.3%) died during their hospitalization which the CRKP infection contributed to their death (Table 1).

### Antimicrobial susceptibility testing and phenotypic assays

Overall the antibiotics showing the highest susceptibility were tigecycline (*n* = 31, 100%), colistin (*n* = 30, 96.8%), amikacin (*n* = 27, 87.1%) and fosfomycin (*n* = 21, 67.7%). MIC range (mg/L) for different carbapenems were imipenem 1- ≥ 256, meropenem 4- ≥ 256 and ertapenem 0.5 - ≥ 256. Seven isolates had MIC ≤8 mg/L for at least one carbapenem. PCR and sequence analysis of carbapenemase genes identified bla_NDM-1_ in 14 isolates and bla_KPC-2_ in 5 isolates. However, it was worth noting that, eight isolates were positive in mCIM combined eCIM while were negative by PCR (Table 1).

### Microbiological investigation of the outbreak

Of the 31 CRKP isolates, 20 (64.5%) were found to produce carbapenemases. However, 6/31 *K. pneumoniae* isolates were found to harbor genes encoding ESBLs (mainly CTX-M-17 and CTX-M-3), whereas only one harbored AmpC genes (DHA-6). Only one strain was found to express 2 types carbapenemases which were KPC-2 and NDM-1 (Table 1).

**Fig. 3:**
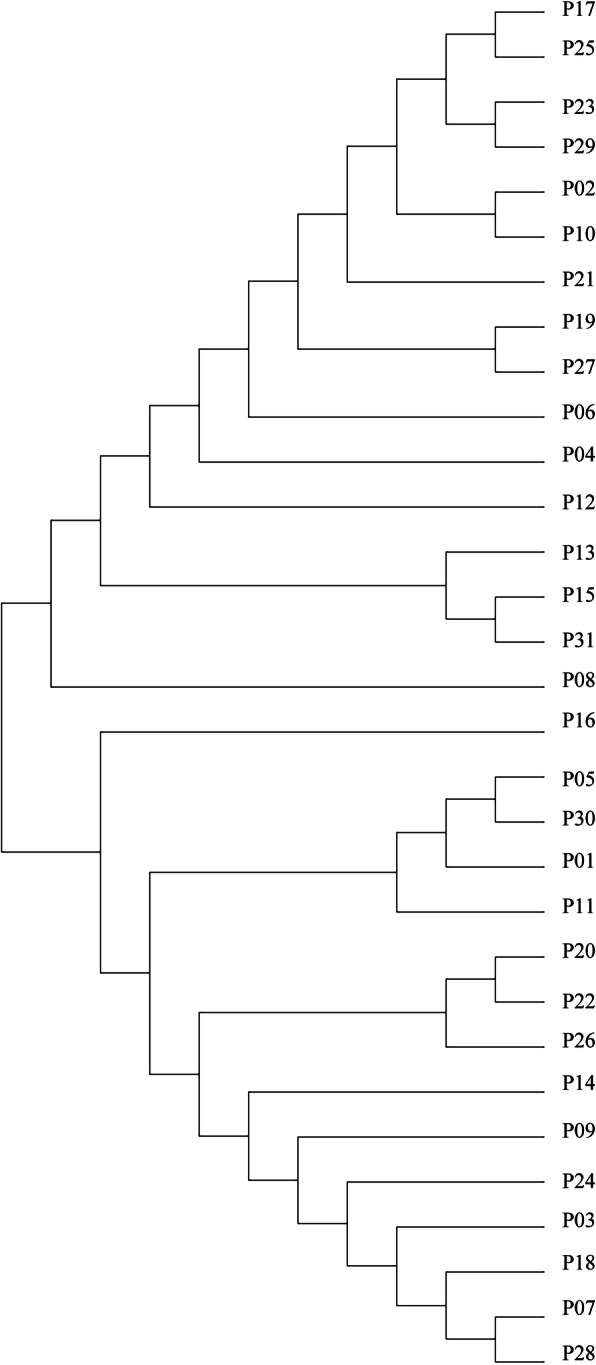
Phylogeny by the seven MLST gene sequences of 31 strains. Patient numbers are shown on the right. The refined maximum likelihood phylogeny constructed by MEGA7. ST: sequence type

The analysis of CRKP revealed a structure dominated by two major genetically distinct lineages; mostly MLST-sequence type belonged to ST11 (21/31) and others such as ST147 (*n* = 1), ST1 (*n* = 1). Different strains producing different carbapenemases that were found in both major related clusters belonging to different STs (Fig. 3).

## Discussion

We report the investigation of the first hospital outbreak of CRKP in the Second Hospital of Shanxi Medical University, which demonstrated extensive clonal dissemination. The epidemiological investigation shown the two index patients had been transferred to regular wards respectively. With frequent transfer of patients between the different wards, the CRKPs were disseminated to ten wards during the outbreak. According to the dendrogram, the isolates were belonging to two closely related clusters with different STs. Horizontal gene transfer via highly transmissible plasmids from CRKP probably occurred.

It is likely that our patients obtained the isolates within wards from an unknown source after admission. The most frequent sources in hospital outbreaks are the patients themselves, the health care staff, or the environment [20], with the former being the most important epidemiologically. Factors such as immunosuppression, ICU admission, antibiotics exposure including carbapenems, invasive devices are making patients at risk of infections by CRKP [21]. The 31 patients involved in the outbreak had complex, extended or repeated, and overlapping inpatient stays, which lead to an increased risk of multidrug resistance strains infection [22, 23]. Mostly of the patients who received Carbapenem-including treatment in the 90 days before the strains isolated. Extended-spectrum antibiotics use could have played a major role in the loss of intestinal microbiota diversity and made CRKP detection possible in subsequent cultures [24–27]. The duration of CRKP colonization is difficult to anticipate and while many patients do experience spontaneous decolonization within 6 months to a year, multiple hospitalizations and CRE disease can extend duration of carriage [28]. Also several studies emphasize the importance of early identification of carriers for infectious control purposes [29, 30]. According to this, the AMS workers asked physicians to submission of active surveillance cultures timely. Invasive devices and ICU admission has been reported that significantly associated with CRKP strains [31–33]. Here we found that 12 of our patients at ICU and 29 of our patients received invasive devices including 5 neurosurgery patients. Thus, our AMS workers have recommended that surveillance culture of feces or rectal swabs or perianal swabs should be performed as soon as possible after hospital admission or risk exposure. Besides our AMS workers have required all patients with cerebrospinal fluid drawn sanitized by using 0.05% chlorhexidine.

The other CRKP sources are the environment, healthcare workers’ and environmental cleaning staffs’ hands, gloves or gowns contaminated by CRKP isolates, especially some high-touch surfaces in the CRKP positive patient zone, and thus increase potential for transmission [34–36]. During the outbreak, transfer of unknown carriers between healthcare workers and environmental contamination probably happened. An indirect epidemiological link for some CRKP-positive cases were likely obtained the strain from environmental source or via undetected intermediate patient. Outbreaks of *K. pneumoniae* with persisting environmental reservoir and high resistance to cleaning efforts have been previously reported [37]. According to this, the AMS workers isolated infected patients in a single room, enhanced chlorhexidine disinfection of the rooms at least three times daily [29, 38] and training sessions in handwashing for healthcare workers.

It also turned out that the infection control methods used previously were not effective in controlling the spread. Our infection control strategies have changed from decentralized management to integrated management now. The assignment of dedicated AMS workers guided by local epidemiology, resource availability and the likely clinical impact of the CRKP outbreak. To support surveillance, enhanced training on epidemiological methods, appropriate data collection and management infrastructure have also implemented. Under the comprehensive management of unified leadership, timely communication, multidisciplinary cooperation and effective surveillance, we successfully controlled the outbreak.

We are now back to epidemiological stage with sporadic CRKP occurrence before the outbreak. The control of CRKPs spread is still possible in hospital settings and relies on the use of rapid diagnostic techniques and strict implementation of hygiene measures. The assignment of dedicated AMS workers proved to be crucial and a multimodal and multidisciplinary infection control strategy seems to be most effective. A study compared infection control practices among nine neighboring hospitals in New York (NY, USA) and found that hospitals that used active surveillance cultures had most success in reducing the acquisition rate of KPC-positive organisms [39]. Nevertheless, in our study, it is difficult to say which infection control intervention is the most effective. Infection control interventions generally are implemented as bundles, since no one action can be singled out as effective [5].

The China Antimicrobial Resistance Surveillance Report (http://www.carss.cn/), the largest survey of antimicrobial resistance in China, reported that the rate of carbapenem resistance in *K. pneumoniae* increased from 8.7% in 2016 (including 1412 tertiary-care hospital and 378 Second-level hospital) to 10.1% in 2018(including 1353 tertiary-care hospital and 349 Second-level hospital). Fortunately, despite the increasing CRKP prevalence recently in our hospital, cross-species transfer of blaNDM-1 and blaKPC-2 from *K. pneumoniae* to other *Enterobacteriaceae* species, particularly *Escherichia coli*, was not detected during the outbreak. CRKP strains harboring KPC are prevalent in China [40], the United States, Israel, Romania, Greece, Italy, and some parts of the Mediterranean region [41]. According to a CRE network to investigate the epidemiology of CRE in China starting from 2014, only CRKP from the Northwest produced a high level of NDM [40]. Our study showed that NDM-1 (15/31) was the main mechanism of carbapenem resistance during the outbreak. Currently, contact with endemic countries, either by tourism or by healthcare, is considered the main risk factor for NDM-1 acquisition [42, 43]. Our patients did not have a history of travel outside of China, but a number had frequent hospitalizations in Shanxi province, raising concerns regarding the possibility of increasing but unrecognized prevalence of NDM-1 and potential decline in value of travel history a marker of colonization risk. Although 11 strains were negative by PCR, they were highly suspected to be other untested carbapenem enzymes. In Asia, the dominant clone is ST11 CRKP, which accounts for up to 60% of CRKP in China [44]. Consequently, in recent years, ST11 has been regarded as the most transmissible clone contributing to the increasing prevalence of CRKP in China [40]. Our study showed that ST11 was the most abundant *K. pneumoniae* ST type. Most ST11 CRKP isolates carried NDM-1 carbapenemase.

There have been a few effective drugs to treat CRKP infections, such as ceftazidime-avibactam and colistin. The new antimicrobial drug ceftazidime-avibatan can be used to treat CRKP which has no metalloenzyme [45], and therefore the detection of genotype is also necessary. During the outbreak, Clinical pharmacists put forward suggestions on rational drug use according to the result of phenotypic assays. In cases where there is a lack of effective drugs, combinations of two or more antibiotics are often used in the hopes of achieving a synergistic effect. The Result of mCIM combine with eCIM showed NDM was the main mechanism of carbapenem resistance during the outbreak. NDM-1 confers broad spectrum beta-lactam resistance mediated by hydrolysis of all β-lactam antimicrobials, with the exception of monobactams, such as aztreonam [9]. Therefore, our AMS workers used tigecycline or colistin in combination with aztreonam for patients with NDM strains in controlling the outbreak.

This study have several limitations. The transmission chain can be further elucidated with a detailed epidemiological investigation, an analysis of plasmids and the use of more discriminatory genotyping such as whole-genome sequencing or pulsed-field gel electrophoresis. Unfortunately, it’s impossible for us now due to the limitation of experimental conditions. Another limitation of our study is the lack of active surveillance cultures results that could probably give useful information about the mode of transmission and the colonization burden of the outbreak-strain. Further work is needed to determine the importance of environmental contamination with CRKP and the effect of effective decontamination on hospital infection rates. Finally, the other limitation of our study was retrospective design.

## Conclusions

This study describes an outbreak of 31 CRKP strains presumably cross-transmitted among patients hospitalized in our hospital from October 2017 to August 2019. The assignment of dedicated AMS workers proved to be crucial. The multimodal and multidisciplinary infection control strategies such as strict contact precautions, isolation, individualized therapy, a comprehensive educational campaign at all levels and strong management seems to be most effective for successful control of the outbreak. The emergence of CRKP in our hospital strengthens the importance of continuing active surveillance cultures, including healthcare workers, common environmental areas and jointly used medical devices on these isolates, thus would help limiting spread of CRKP and improving management.

## Data Availability

The datasets used and analysed during the current study are available from the corresponding author on reasonable request.

## Abbreviations

CRKP: Carbapenem-resistant *Klebsiella pneumoniae*
AMS: Antimicrobial stewardship
mCIM: Modified carbapenem inactivation method
eCIM: EDTA-modified carbapenem inactivation method
PCR: Polymerase chain reaction
MLST: Multilocus sequence typing
MDR: Multidrug resistant
ESBLs: Extended spectrum β-lactamases
CLSI: Clinical and Laboratory Standards Institute
MBLs: Metallo-β-lactamases
APACHE II: Acute Physiology and Chronic Health Evaluation II
*K. pneumoniae*: *Klebsiella pneumoniae*
ICU: Intensive care unit
